# Effect of empagliflozin on copeptin levels in patients with recent acute coronary syndrome and newly detected dysglycaemia: a post-hoc analysis of the SOCOGAMI randomized controlled trial

**DOI:** 10.1186/s12933-026-03312-y

**Published:** 2026-07-28

**Authors:** Elena Fortin, Olle Melander, Giulia Ferrannini, Per Näsman, Anna Norhammar, Lars Rydén, Stina Smetana, Malin Svensson, Linda Mellbin

**Affiliations:** 1 Department of Medicine K2 Solna, Karolinska Institutet, Stockholm, Sweden; 2 Department of Clinical Sciences Malmö, Lund University, Malmö, Sweden; 3 Internal Medicine Unit, Södertälje Hospital, Södertälje, Sweden; 4 Center for Safety Research, KTH Royal Institute of Technology, Stockholm, Sweden; 5 Capio St Görans Hospital, Stockholm, Sweden; 6 Heart and Vascular Theme, Karolinska University Hospital, Stockholm, Sweden

**Keywords:** Empagliflozin, Copeptin, Acute coronary syndrome, Dysglycaemia, Type 2 diabetes

## Abstract

**Background:**

Copeptin, a surrogate marker for vasopressin secretion, is associated with cardiovascular disease, insulin resistance and dysglycaemia. The cardioprotective effects of sodium-glucose cotransporter 2 inhibitors (SGLT2i) may involve vasopressin modulation through fluid redistribution, but whether this effect persists long-term in high-cardiovascular-risk patients with newly detected dysglycaemia remains unknown.

**Methods:**

In this post-hoc analysis of the SOCOGAMI double-blind, placebo-controlled trial, 42 patients (mean age 67.5 years, 19% females) with impaired glucose tolerance or newly detected type 2 diabetes following an ACS and no heart failure were randomized to empagliflozin 25 mg/day (*n* = 20) or placebo (*n* = 22) for 7 months. Copeptin was measured during oral glucose tolerance tests (OGTT) at baseline, after 7 months on-treatment, and 3 months after treatment withdrawal. Treatment effects were assessed by repeated-measures ANOVA with treatment × time interaction and linear mixed-effects models.

**Results:**

Haematocrit, but not copeptin, showed a significant between-group difference at 7 months (*p* = 0.03 and *p* = 0.63, respectively). Both markers returned toward baseline after treatment withdrawal, but the overall treatment × time interaction was not significant for either (*p* = 0.72 and *p* = 0.64 respectively). Results were unchanged after accounting for a baseline imbalance in diuretic use (35% vs. 14%). Copeptin was not associated with the glucose-lowering effect of empagliflozin and no differential copeptin response during the OGTT across groups or visits was observed. In exploratory analyses, copeptin correlated with arterial pulse wave velocity at baseline (rs = 0.40, unadjusted *p* = 0.03).

**Conclusions:**

In this post-hoc analysis, empagliflozin treatment was not associated with statistically significant sustained vasopressin secretion in post-ACS patients with newly detected dysglycaemia and preserved cardiac function. Due to the limited power and the absence of early on-treatment sampling these findings cannot exclude AVP modulation and warrant confirmation in adequately powered studies.

**Trial registration:**

EudraCT number 2015-004571-73.

**Graphical abstract:**

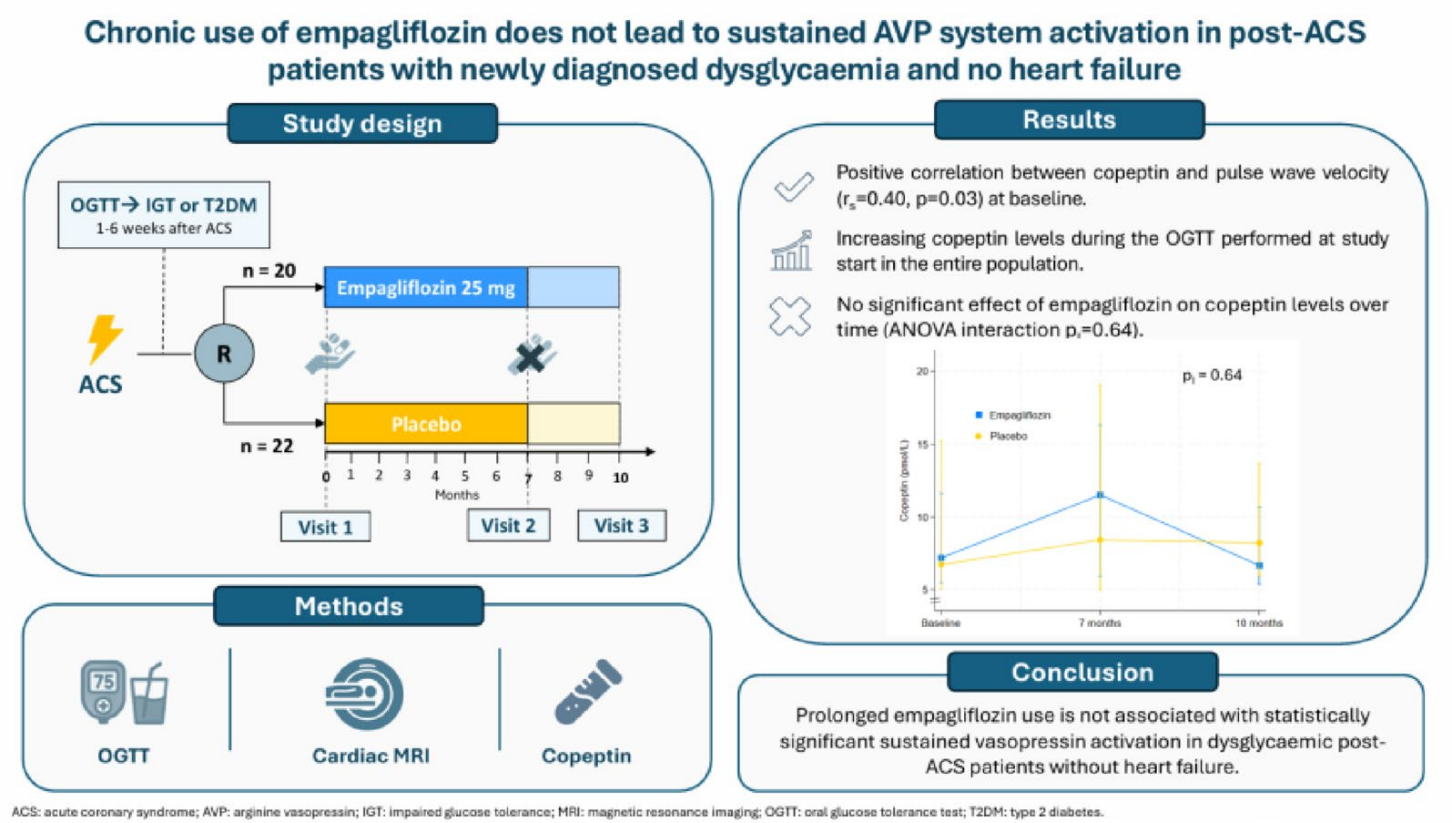

**Supplementary Information:**

The online version contains supplementary material available at 10.1186/s12933-026-03312-y.

## Research insights

**What is currently known about this topic?**

- Copeptin reflects vasopressin secretion and is linked to both dysglycaemia and coronary heart disease.
- SGLT2 inhibitors induce osmotic diuresis and natriuresis, potentially triggering compensatory vasopressin secretion via reduced circulating volume.

**What is the key research question?**

- Are copeptin levels affected by Empagliflozin therapy in patients with newly detected dysglycaemia after acute coronary syndrome?

**What is new?**

- Empagliflozin was not associated with statistically significant sustained AVP secretion in patients with high cardiovascular risk and dysglycaemia.

**How might this study influence clinical practice?**

- These data provide preliminary reassurance against sustained AVP activation during chronic empagliflozin use in post-ACS dysglycaemia.

## Background

Coronary heart disease (CHD) and dysglycaemia often co-exist and their combination is associated with a worse prognosis [1]. Hyperglycaemia-related detrimental effects on myocardial recovery via intensified inflammation, endothelial dysfunction and maladaptive neurohormonal activation are considered of crucial importance for this interaction [2, 3]. The arginine vasopressin (AVP) system has gained increasing attention within the field of cardiometabolic disorders due to its regulatory influence on cardiovascular homeostasis and its role in glucose and insulin metabolism [4, 5]. Due to its rapid clearance from the circulation and its ex-vivo instability, AVP is routinely assessed via copeptin, a stable cleavage product derived in equimolar amounts to AVP from the C-terminal part of its prohormone [6]. Altered copeptin levels have been documented across a wide range of cardiovascular conditions, including coronary artery disease and heart failure (HF), where AVP activation is of interest due to its independent prognostic capacity [7–10]. Elevated copeptin is associated with impaired insulin sensitivity and an increased risk of developing diabetes [11, 12]. While AVP may influence glucose homeostasis through insulin, glucagon and ACTH regulation, the direction of causality remains uncertain. Sodium-glucose cotransporter-2 inhibitors (SGLT2i), such as empagliflozin, have cardiorenal protective effects in populations with and without diabetes [13, 14]. Recent clinical studies have indicated that SGLT2i-induced osmotic diuresis activates an adaptive mechanism involving the AVP-renal axis to maintain fluid homeostasis, as evidenced by increased copeptin levels already after a few weeks of treatment [15, 16]. Whether this effect persists over time or applies even in patients with newly detected dysglycaemia, is, however, unknown.

In this post-hoc analysis of the SOdium-glucose CO-transporter inhibition in patients with newly detected Glucose Abnormalities and a recent Myocardial Infarction (SOCOGAMI) trial we aimed to provide novel insights on the mechanistic effects of SGLT2 inhibition by exploring its effects over time on AVP signalling in relation to glycaemic control in patients with newly detected impaired glucose tolerance (IGT) or type 2 diabetes (T2DM) in a post-acute coronary syndrome (ACS) setting.

## Methods

### Trial design and study protocol

The SOCOGAMI Study is a single-site, randomized, double blind, placebo-controlled trial conducted at the Cardiology Unit of the Department of Medicine K2 at Karolinska Institutet, Sweden March 2016 to June 2018. The primary objective was to explore the effects of SGLT2i on myocardial function and structure by means of cardiac magnetic resonance (CMR) and on beta cell function, in patients with newly discovered IGT and T2DM after a recent ACS. The trial protocol, including 5 visits over a 10-month period, and previous substudies have previously been described in detail [17, 18].

In brief, the study enrolled adults who (1) had experienced an ACS, i.e. an acute myocardial infarction (AMI) or unstable angina pectoris as defined by joint European and American guidelines [19], and (2) in connection with the ACS had been diagnosed with dysglycaemia defined as IGT or T2DM. Main exclusion criteria were known diabetes, an estimated glomerular filtration rate (eGFR) < 30 ml/min/1.73 m^2^ and heart failure. At a baseline visit 6–10 weeks after the ACS all participants underwent a physical examination, an OGTT and CMR imaging whereafter they were randomized to empagliflozin 25 mg daily or placebo. Seven months later all baseline assessments, including OGTT and CMR, were repeated. Following this the study drug was discontinued and the participants returned after three months (i.e., ten months after randomization) and the full set of investigations was repeated. After the 7-month visit the study drug was discontinued and a 3-month drug-free interval preceded the 10-month visit, by which time the drug had cleared. No formal washout medication protocol was applied. Although the original SOCOGAMI protocol comprised five visits over ten months, the full OGTT and copeptin sampling protocol was performed only at the three main study visits (baseline, 7 months on-treatment, and 10 months, i.e. 3 months after withdrawal).

The trial was carried out in compliance with the principles of the Declaration of Helsinki. All participants gave their informed consent. The study was approved by the local ethics committee (Dnr 2015/1870-31/4), the Swedish Medical Drug Agency (EU-nr 2015-004571-73) and had the following trial registration number EudraCT number 2015-004571-73.

### Procedures and definitions

The *OGTT* was performed following an overnight fast. Plasma glucose was measured before and 30 and 120 min after glucose administration (75 g in 200 ml water) by means of the HemoCue® Glucose 201 RT (HC201RT) equipment. Glucose levels obtained during the OGTT were used to confirm the dysglycaemic state of the study participants at baseline and at the following study visits according to criteria by the World Health Organization (WHO) [20].

*Plasma copeptin* concentrations at baseline and during the OGTT at the three main visits (baseline, 7 months and 10 months) were determined by use of BRAHMS Copeptin proAVP Kryptor immunofluorescence assay on the Kryptor Gold analyzer (Thermo Fisher Scientific, BRAHMS GmbH, Hennigsdorf, Germany). The assay had a lower limit of detection of 0.90 pmol/L and a functional assay sensitivity (< 20% inter-assay coefficient of variation) of 1.08 pmol/L.

*Plasma insulin* and *C-peptide* were measured at all three visits and OGTT time points and just in the fasting state, respectively, through an electrochemical luminescence immunoassay (ECLIA, Roche, reference interval for adults in the fasting state 2.0–25 mU/L and 0.5–1 nmol/L, respectively) and used to calculate insulin resistance indexes. The Homeostatic Model Assessment of insulin resistance (HOMA-IR) was defined as fasting insulin (mU/L) × fasting glucose (mmol/L)/22.5 [21]. The metabolic clearance rate stumvoll index (MCR Stumvoll) [22] was calculated by the following formula:

$$ \begin{aligned} & 19.240 - 0.281 \times {\mathrm{BMI}}_{{{\mathrm{kg/m}}^{{2}} }} - 0.00498 \\ & \quad \times {\mathrm{Ins}}120_{{\mathrm{pmol/L}}} - 0.333 \times {\text{ Glu120}}_{{\mathrm{mmol/L}}} \\ \end{aligned} $$

The *CMR* procedure has been described in detail elsewhere [17]. In brief, CMR at 1.5 T was performed with adenosine stress testing and a first pass perfusion imaging using Gadobutrol as intravenous contrast agent. Left ventricular volumes, systolic function, stroke volume, and mass were assessed by cine, steady-state free precession imaging. Arterial pulse wave velocity (aPWV) was derived from through-plane two-dimensional phase-contrast flow acquisitions in the ascending and abdominal aorta. Transit time was calculated using the flow-curve foot (time-to-foot) method, and aortic path length was measured along the vessel centre line between the two planes, using Segment CMR [23].

## Research resource identifiers (RRIDs)

Key resources used in this study are identified by Research Resource Identifiers: BRAHMS Copeptin proAVP KRYPTOR assay (RRID: AB_3073917); Stata/MP 18.0 (RRID:SCR_012763).

### Statistical methods

Baseline characteristics are presented as mean ± standard deviation (SD) for continuous variables following a normal distribution or as median [1st and 3rd quartile (Q1, Q3)] for skewed distributions, as determined by the Shapiro–Wilk test. Categorical data are reported as frequencies and percentages. Differences in baseline characteristics between empagliflozin vs. placebo were analyzed using the Student’s t-test or the Mann–Whitney U-test for continuous variables, depending on distribution, and by the χ^2^ test for categorical variables. Differences across OGTT timepoints at baseline were investigated by Friedman test and Wilcoxon signed-rank test for pairwise analyses. Correlations between copeptin levels at baseline, and selected variables in the total cohort were evaluated using the Spearman correlation coefficient.

The impact of treatment (empagliflozin/placebo) on copeptin and haematocrit (HCT) levels across the three study visits was primarily investigated by separate repeated measures (RM) ANOVA models. These models included a treatment group x time interaction term to examine differences of log-transformed copeptin and haematocrit over time. The sphericity assumption for the ANOVA models was evaluated using Mauchly´s W test and appropriate corrections were applied if necessary. To address potential limitations of RM-ANOVA two different sensitivity analyses were conducted. First, a linear regression model was applied in a subset of patients with complete data at baseline and at 7 months (*n* = 35), with Δ values (7 months—baseline) and treatment group as dependent and independent variable, respectively, adjusting for baseline copeptin levels to control for inter-individual differences. Second, to allow for more flexibility in handling missing data and modelling within-subject correlations across both visit- and OGTT-timepoints, a linear mixed-effects model was applied. This model was estimated using Restricted Maximum Likelihood (REML) and aimed to assess copeptin variations across treatment groups (empagliflozin/placebo), study visits (baseline, 7 months, 10 months) and OGTT timepoints (0, 30, 120 min) incorporating random intercepts for participants to account for inter-individual variability as well as fixed effects for treatment group, visit, OGTT time, and their interactions.

Additional linear regression models were used to test whether baseline copeptin levels would predict the significant glucose-lowering effect of empagliflozin, both in the empagliflozin-treated group alone and in both groups together, including a copeptin x treatment allocation interaction term in the latter case. Given the presence of outliers, robust linear regression was used in this case.

Of 42 randomized patients, one had no copeptin data at any visit and two contributed a single observation each; these three patients were excluded, leaving 39 patients for the primary repeated-measures ANOVA and mixed-effects model analyses. Baseline copeptin was unavailable in 5 patients (empagliflozin *n* = 3, placebo *n* = 2; Fisher’s exact *p* = 0.66), leaving 35 patients with complete data at baseline and 7 months (used for the sensitivity complete case analysis). No values were imputed. Per-visit data availability is reported in Supplementary Table 1.

The trial was not originally designed for the copeptin endpoint. Given the observed 7-month standard deviation (6.75 pmol/L) and sample size, the minimum between-group difference detectable at 80% power (two-sided *α* = 0.05) was 6.4 pmol/L.

Statistical significance was considered for a two-sided *p* < 0.05. All statistical analyses were performed using STATA/MP 18.0. The SOCOGAMI trial was conducted and reported in accordance with the CONSORT guidelines.

## Results

### Baseline characteristics of the trial population

A total of 42 patients (mean age 67.5 years, 19% females; AMI = 36; unstable angina = 6; IGT = 27; T2DM = 15) were randomized to either empagliflozin 25 mg/day (*n* = 20) or placebo (*n* = 22). The two groups were well balanced as regards all demographic and clinical characteristics, including BMI, glycaemic group (60% IGT and 40% T2DM in the empagliflozin group versus 68% IGT and 32% T2DM in the placebo group), eGFR, electrolytes, HCT, NT-proBNP, and medications (Table 1). No participant was on any glucose lowering medication at study start. The use of diuretics at baseline was low: 7 patients in the empagliflozin group *vs.* 3 in the placebo group. There were no changes in the diuretic dose during follow-up but for an increase in one of the empagliflozin patients (Supplementary Table 2).

**Table 1:** Overview of baseline patients characteristics by allocated group

Variable	Empagliflozin (*n* = 20)	Placebo (*n* = 22)	Missing
Age (years)	67 (8)	68 (8)	0
Male sex	16 (80%)	18 (82%)	0
Waist circumference (cm)	100 (95, 105)	102 (95, 108)	6
BMI (kg/m^2^)	27.0 (4.1)	27.1 (4.2)	0
Eligibility criteria			
Index event (MI/UA)	17/3	19/3	0
IGT/T2DM	11/9	14/8	0
Medical history			
Prior TIA/Stroke	2 (10%)	0 (0%)	0
Peripheral artery disease	1 (5%)	0 (0%)	0
Heart failure	1 (5%)	0 (0%)	0
Known family history of CVD**	5 (26%)	9 (45%)	3
Known family history of T2DM**	5 (28%)	6 (29%)	3
Smoking habits			0
Current	1 (5%)	7(32%)	
Previous (> 1 month)	14 (70%)	12 (55%)	
Never	5 (25%)	3 (14%)	
Blood pressure (mmHg)			
Systolic	130 (16)	131 (16)	0
Diastolic	80 (74, 85)	80 (77, 85)	0
Laboratory values			
LDL-C (mmol/L)	1.43 (0.35)	1.43 (0.59)	1
HDL-C (mmol/L)	1.24 (0.34)	1.18 (0.38)	1
Creatinine (µmol/L)	85.9 (15.6)	81.1 (18.4)	1
eGFR (ml/min/1.73 m^2^)	68.2 (12.6)	72.9 (14.0)	2
Haemoglobin (g/L)	141 (135, 150)	142 (135, 149)	1
Haematocrit (%)	43 (40, 44)	43 (40, 45)	2
Troponin (ng/L)	11.0 (9.0, 14.0)	11.5 (10.0, 21.0)	1
Triglycerides (mmol/L)	1.0 (0.9, 1.4)	1.2 (1.0, 1.4)	1
hs-CRP (mg/L)	1.1 (0.6, 1.7)	1.1 (0.7, 1.4)	1
NT-proBNP (ng/L)	143 (72, 514)	156 (62, 236)	1
FPG (mmol/L)	6.2 (6.0, 7.2)	6.3 (6.0, 6.7)	0
2 h-PG (mmol/L)	10.7 (8.7, 12.2)	9.7 (8.6, 12.3)	1
HbA1c (mmol/mol)	41 (39, 45)	42 (40, 47)	2
Copeptin (pmol/L)	7.18 (5.46, 11.62)	6.72 (5.09,15.21)	5
Pharmacological treatment			
ACE inhibitors/ARBs	17 (85%)	18 (82%)	0
ARNi	0 (0%)	0 (0%)	0
β-blockers	17 (85%)	21 (95%)	0
Ca-blockers	5 (25%)	4 (18%)	0
Loop/tiazid diuretics	7 (35%)	3 (14%)	0
MRA	2 (11%)	0 (0%)	1
Statins	20 (100%)	21 (96%)	0
Aspirin	19 (95%)	21 (95%)	0

Continuous data are either mean (SD) for normally distributed variables (age, BMI, blood pressure, LDL-C, HDL-C, creatinine, eGFR) and median (Q1, Q3) for skewed variables (waist circumference, haemoglobin, haematocrit, troponin, triglycerides, hs-CRP, NT-proBNP, glycaemic variables, copeptin). Categorical data are *N* (%). **Defined as a close relative with CVD or T2DM at < 60 years of age and based on self-reported information in standardized questionnaires

MI, myocardial infarction; BMI, body mass index; UA, unstable angina; TIA, transitory ischaemic attack; IGT, impaired glucose tolerance; T2DM, type 2 diabetes; LDL-C, low-density lipoprotein; eGFR, estimated glomerular filtration rate; hsCRP, high-sensitivity C-reactive protein; BNP, brain natriuretic peptide; HbA1c, glycated haemoglobin A1c; ACE, angiotensin converting enzyme; ARB, angiotensin receptor blocker; ARNi, angiotensin receptor/neprilysin inhibitor; β-blockers, Beta blockers; Ca-blockers, Calcium channel blockers; MRA, mineralcorticoid antagonists

Table 2 and Supplementary Fig. 1 illustrate that copeptin levels were similar in IGT and T2DM patients, both at fasting (IGT vs. T2DM: 6.78 (4.49, 14.70) pmol/L versus 7.12 (6.57, 11.57) pmol/L, *p* = 0.65) and during the OGTT (Δcopeptin_120-0_ IGT vs. T2DM: − 0.88 (− 4.07, 0.19) pmol/L vs. − 0.90 (− 2.06, 1.41) pmol/L, *p* = 0.52). Consequently, the two glycaemic groups were treated as a single entity for subsequent analyses.

**Table 2:** Copeptin levels during the OGTT at baseline in all patients and differences between glycaemic groups

	All	IGT	T2DM	*p*† IGT versus T2DM
OGTT time				
Copeptin 0 min	7.05 (5.18, 14.68)	6.78 (4.49, 14.70)	7.12 (6.57, 11.57)	0.65
Copeptin 30 min	7.35 (4.89, 10.26)	6.87 (4.46, 11.46)	8.68 (5.14, 9.74)	0.76
Copeptin 120 min	8.52 (4.74, 11.49)	7.31 (4.29, 13.48)	9.92 (4.86, 10.76)	0.69
Copeptin Δ_120-0_	− 0.91 (− 3.24, 0.26)	− 0.88 (− 4.07, 0.19)	− 0.91 (− 2.06, 1.41)	0.52
Comparisons during OGTT				
p* 0–30–120	< 0.01	< 0.01	< 0.01	
p** 0–30	0.04	0.14	0.15	
p** 30–120	0.70	0.31	0.53	
p** 0–120	0.05	0.07	0.40	

Data are median (Q1–Q3)

^*^p for Friedman test

^**^Wilcoxon signed-rank test

^†^*p* for Mann–Whitney U test

IGT, impaired glucose tolerance; T2DM, type 2 diabetes; OGTT, oral glucose tolerance test

**Fig. 1:**
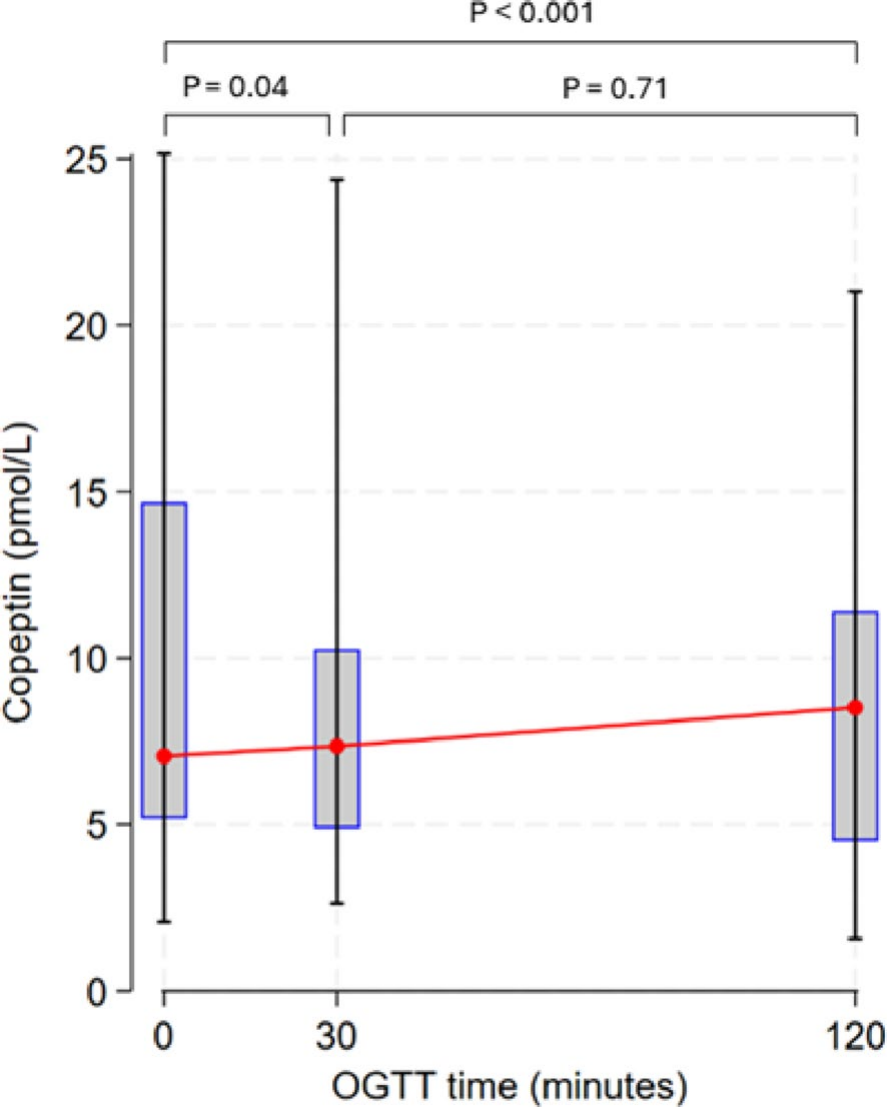
Copeptin levels during the OGTT (0, 30 and 120 min) at baseline. *P*-values are by Wilcoxon signed-rank test. OGTT: oral glucose tolerance test

As shown in Table 2 and Fig. 1, median copeptin levels in the entire population at study start increased during the OGTT from 7.05 (5.18, 14.68; *n* = 37) pmol/L in the fasting state to 7.35 (4.89, 10.26) pmol/L after 30 min, and then went up to 8.52 (4.74, 11.49; *n* = 35) pmol/L 2 h after the glucose load (p_0-30–120_ < 0.01). The median within-patient change from 0 to 120 min (*n* = 33) was − 0.91 pmol/L as most patients declined modestly, while a minority raised the cross-sectional 120-min median (21/33 decreased, 12 increased; Supplementary Fig. 2).

Pairwise comparisons revealed a significant rise from 0 to 30 min after the glucose ingestion (*p* = 0.04), which stabilized thereafter (p_30-120 min_  = 0.71).

### Relationship between copeptin levels and anthropometric, laboratory and CMR variables in all participants before and after treatment

**Fig. 2:**
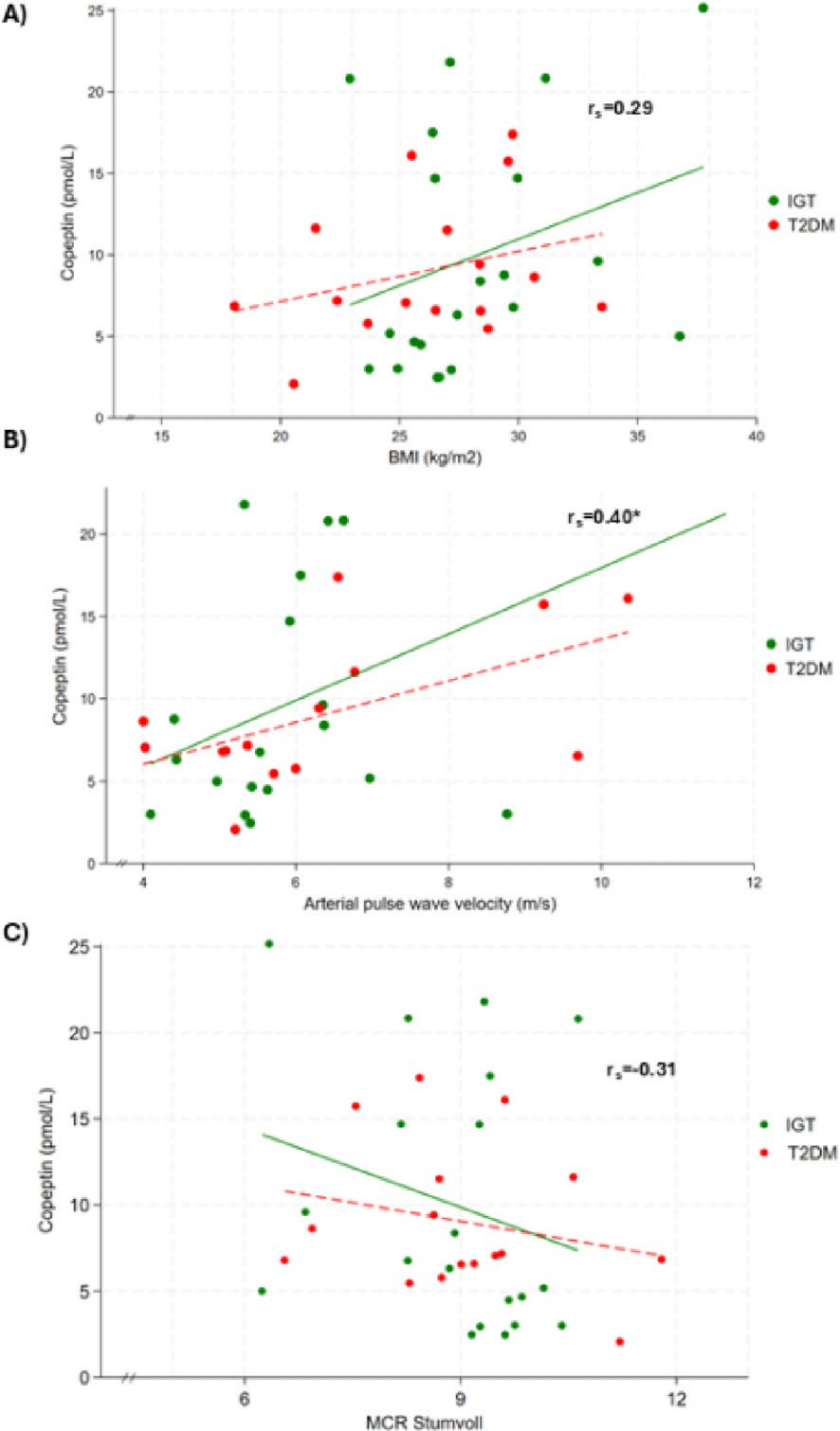
Correlations between copeptin and **A** BMI, **B** arterial pulse-wave velocity and **C** MCR Stumvoll index in patients with IGT (green) and T2DM (red). r_s_ = Spearman correlation coefficient. *r_s_ *p*-value < 0.05. BMI, body mass index; IGT, impaired glucose tolerance; T2DM, type 2 diabetes; MCR, metabolic clearance rate

Correlations between copeptin concentrations and different anthropometric, laboratory and CMR variables at baseline are shown in Table 3 and Fig. 2. The aPWV was the only variable which significantly correlated to the copeptin levels. This correlation was moderate (r_s_ = 0.4, *p* = 0.03). BMI and MCR Stumvoll showed no statistically significant correlation (r_s_ = 0.3, *p* = 0.09 and r_s_ = − 0.3, *p* = 0.07, respectively) with copeptin levels at baseline, as well as HCT, electrolytes or any of the glycaemic variables, i.e. fasting plasma glucose (FPG), 2-h post-load glucose (2hPG) and HbA1c.

**Table 3:** Correlation coefficients of copeptin with anthropometric and glycaemic parameters in the whole cohort at baseline

Variables	Copeptin	
	r_s_	*p*-value
Age	0.22	0.18
BMI	0.29	0.09
HbA1c	0.23	0.18
SBP	− 0.20	0.22
FPG	0.06	0.73
2 h-PG	0.17	0.31
HOMA-IR	0.15	0.40
MCR Stumvoll	− 0.31	0.07
LVSVi	− 0.29	0.11
LVEDVi	− 0.10	0.60
LVESVi	− 0.10	0.62
LVEF	0.03	0.90
aPWV	0.40	0.03
ECV	0.05	0.78
LVMi	0.24	0.18
Na	− 0.16	0.36
K	0.17	0.34
HCT	0.009	0.95

r_s_, Spearman correlation coefficient

BMI, body mass index; LDL-C, low-density lipoprotein; eGFR, estimated glomerular filtration rate; hsCRP, high-sensitivity C-reactive protein; HbA1c, glycated haemoglobin A1c; SBP, systolic blood pressure; FPG, fasting plasma glucose; 2 h-PG, 2-h post-load glucose; HOMA-IR, Homeostatic assessment model-insulin Resistance); MCR, Metabolic clearance rate; LVSVi, left ventricular stroke volume index; LVEDVi, left ventricular end-diastolic volume index; LVESVi, left ventricular end-systolic volume index; LVEF, left ventricular ejection fraction; aPWV, arterial pulse wave velocity; ECV, extracellular volume; LVMi, left ventricular mass index; Na, Sodium; K, Potassium; HCT, haematocrit

After 7 months of treatment, there was no correlation between changes in copeptin levels and glycaemic variables, or HOMA-IR and MCR Stumvoll. A moderate positive correlation was observed between changes in sodium levels in both groups (*p* = 0.02), but no correlations were observed with other metabolic, hydro-electrolyte and CMR parameters (Supplementary Table 3).

### Effects of treatment on copeptin levels and hematocrit

#### Primary analysis

Copeptin concentrations did not differ between the two treatment groups at baseline (empagliflozin vs. placebo: 7.18 (5.46, 11.62) pmol/L and 6.72 (5.09, 15.21) pmol/L, *p* = 0.78). During follow-up, the group treated with empagliflozin showed a reduction in FPG, 2hPG, insulin resistance indexes and BMI, while, as already reported, there was no effect on cardiac structure and function [17].

**Fig. 3:**
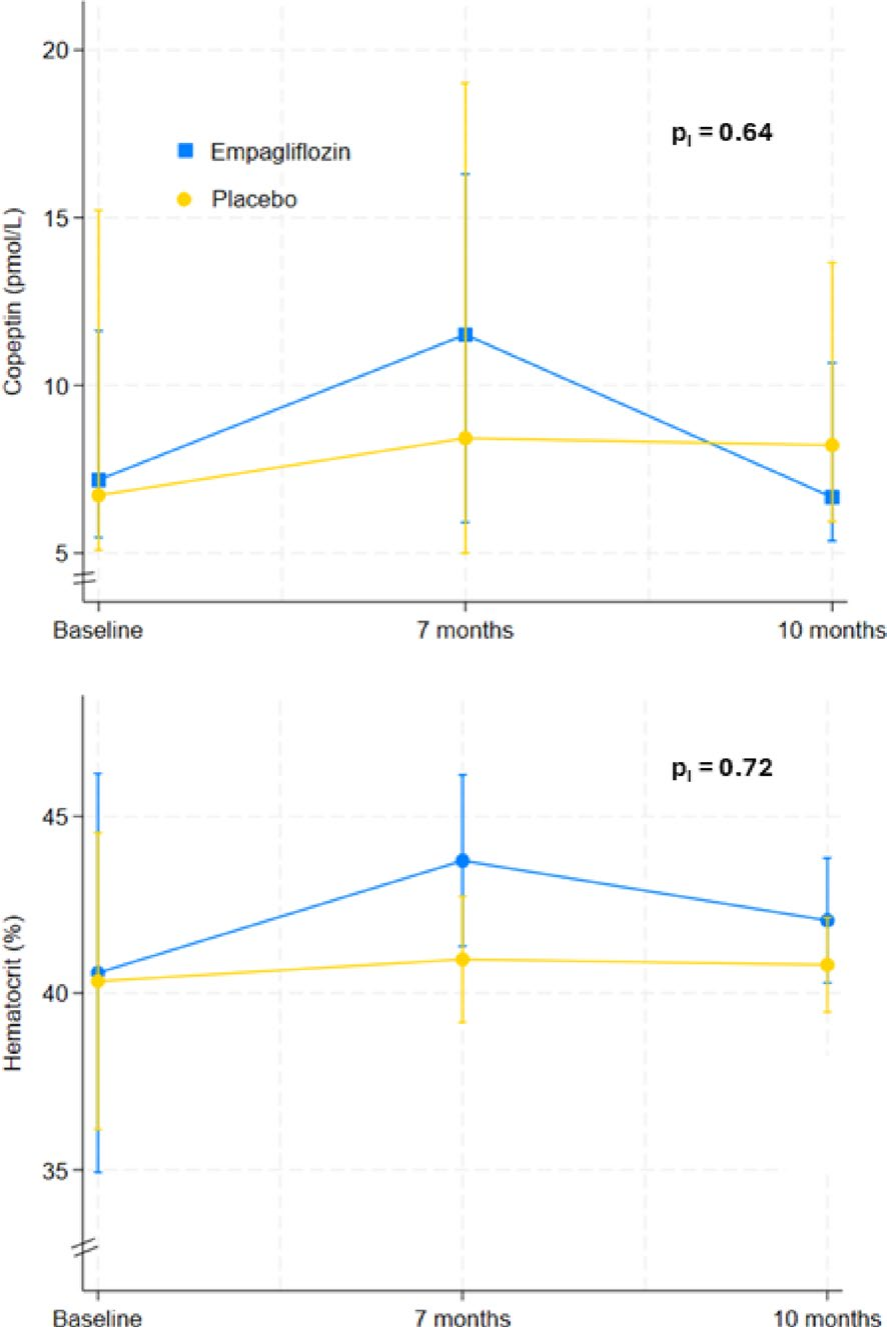
Effect of Empagliflozin (blue) versus Placebo (yellow) on Copeptin (**A**) and Haematocrit (**B**) over time. P_I_ is the *p*-value for interaction between treatment allocation and time of visit in the ANOVA repeated measures model.

To explore the relationship between copeptin and fluid balance, variations of fasting copeptin levels and HCT by treatment group over the entire follow-up period were investigated and are shown in Fig. 3 and Table 4. A non-significant trend towards increased copeptin levels after 7 months was followed by a return to baseline levels after treatment discontinuation in the empagliflozin group compared to placebo (p for interaction between treatment allocation and time of visit in the repeated measures ANOVA model was 0.64). HCT was significantly higher in the empagliflozin group than in the placebo group after 7 months (*p* = 0.03), but this difference was no longer present after treatment withdrawal, and overall treatment × time interaction was non-significant (*p* = 0.72). These results did not change after accounting for diuretic use at baseline and/or diuretic dose changes during the treatment period (*p* = 0.78 for copeptin).

**Table 4:** Copeptin and haematocrit levels by treatment group over the three timepoints of the study

	Baseline			7 months			10 months			*P*_I__overall_
	Empa	Placebo	P*	Empa	Placebo	P*	Empa	Placebo	P*	
Copeptin	7.18 (5.46, 11.62)	6.72 (5.10, 15.21)	0.78	11.51 (5.92, 16.30)	8.43 (5.00, 19.01)	0.63	6.67 (5.36, 10.67)	8.22 (5.94, 13.66)	0.50	0.64
Haematocrit	43 (40, 44)	43 (40, 45)	0.84	44 (41.5, 47)	42 (39, 43)	**0.03**	42.5 (40, 45)	40 (39, 43.5)	0.25	0.72

Values are median (Q1–Q3). P* are *p*-values by Mann–Whitney U test. *P*_I overall_ is *P* for interaction between treatment allocation and time of visit in the whole ANOVA repeated measures model. Bold indicates statistical significance (P < 0.05).

**Fig. 4:**
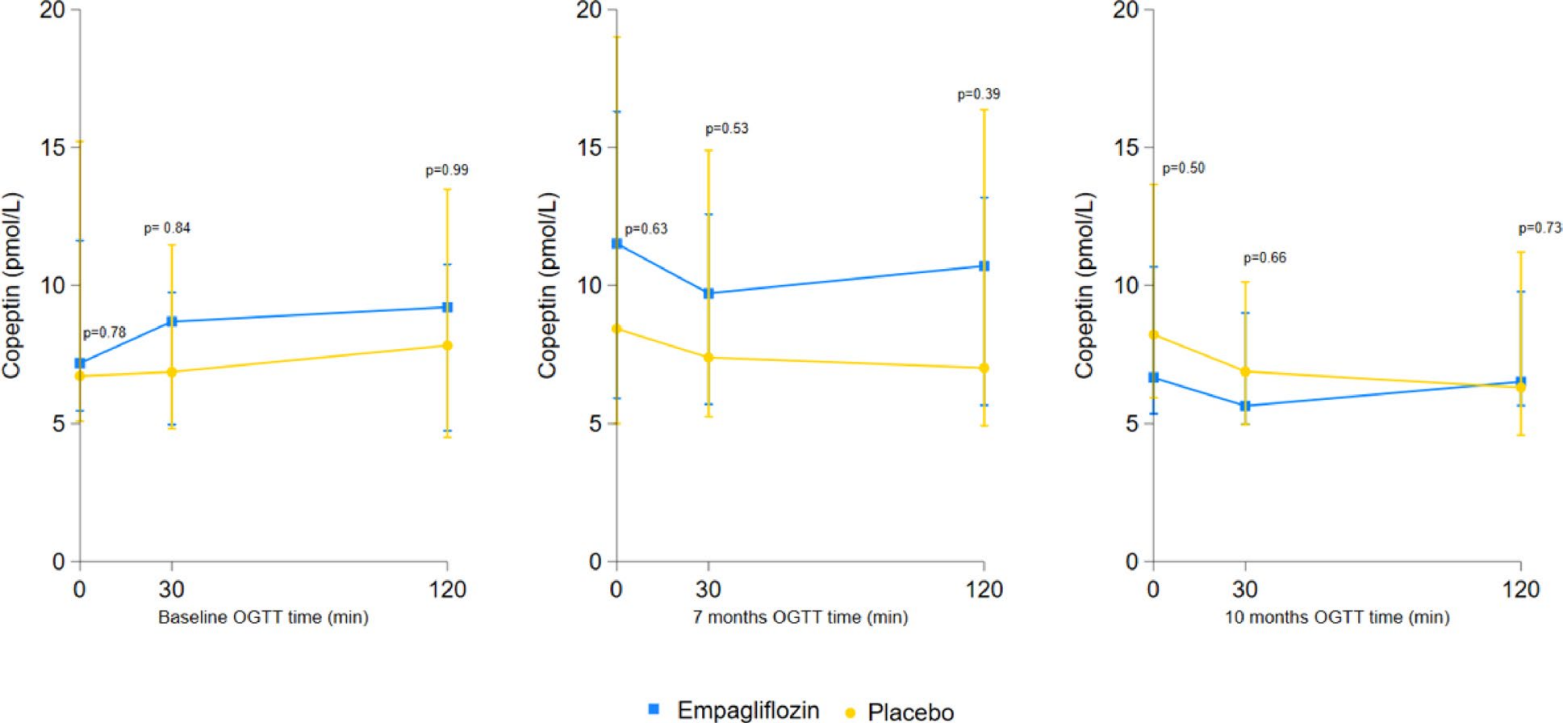
OGTT-related copeptin levels in empagliflozin (blue) and placebo (yellow) at baseline and during follow-up. *P*-values are for differences between empagliflozin and placebo groups at each OGTT timepoint (by Mann–Whitney U Test). OGTT, Oral glucose tolerance test

As shown in Fig. 4 and Supplementary Table 4, there were no statistically significant differences between the two treatment groups in copeptin levels during the glucose challenge across the entire study duration (from baseline to 10 months) when considering each OGTT-timepoint separately.

#### Sensitivity analysis

The evaluation of Δcopeptin levels (from baseline to 7 months) in patients with complete data (empagliflozin *N* = 17 and placebo N = 18) showed slightly higher concentrations in the empagliflozin group versus placebo (+ 1.68 ± 6.04 pmol/L vs. + 0.80 ± 4.27 pmol/L, Supplementary Fig. 3), even after adjustment for baseline copeptin concentrations (adjusted *p* = 0.64). Similarly, ΔHCT increased more after 7 months in the empagliflozin group compared to placebo, but without reaching statistical significance (+ 2.81 ± 9.9% vs. + 0.61 ± 10.5%, *p* = 0.50).

In the linear mixed-effects model analysis, the main effect of treatment was not significant (*p* = 0.95), but the results indicated a small, though not significant, increase in copeptin at 7 months compared to baseline (*p* = 0.08), and no significant change at 10 months (*p* = 0.81). However, there was no treatment group x study visit interaction (all *p* > 0.05). The main effect of the OGTT-time was not statistically significant either, nor was the treatment group × study visit × OGTT-time interaction (all *p* > 0.05), suggesting that empagliflozin did not differentially alter the pattern of copeptin responses across OGTT-timepoints.

### Copeptin value for empagliflozin glucose-lowering effect

Baseline copeptin levels had no predictive value for the magnitude of the glucose-lowering effect of empagliflozin neither in the fasting nor in the post-load state (*p*-values = 0.82 and 0.66, respectively). Moreover, there was no evidence that the relationship between baseline copeptin and the change in glucose levels (either at fasting or post glucose challenge) from pre- and post-treatment would depend on treatment group (*p*-values for the copeptin × treatment group interaction term on top on main effect in the regression models = 0.47 for ΔFPG and 0.80 for Δ2h-PG).

## Discussion

To the best of our knowledge, this is the first evaluation of copeptin response to SGLT2i in a well-characterized group of post-ACS individuals with newly detected dysglycaemia and without symptoms of HF or reduced ejection fraction, providing new insights into SGLT2i-mediated AVP-system modulation. In this exploratory post-hoc analysis, we were unable to detect a statistically significant effect of empagliflozin on AVP secretion.

Our results partially contrast prior studies reporting significant copeptin increases after 1 and 3 months of SGLT2i use in patients with T2DM and/or HF [16, 24, 25]. The observed trend toward increased copeptin levels in the empagliflozin group at 7 months from 7.18 pmol/L to 11.51 pmol/L in our population, followed by normalization after treatment discontinuation is in range with data recently reported by Berton et al. in a population with T2DM [16]. Unlike studies restricted to T2DM, SOCOGAMI included patients with newly detected IGT and T2DM, representing an early stage of disease. Although the two glycaemic groups did not differ significantly in copeptin levels, the inclusion of IGT patients may have introduced inter-individual variability in AVP, as confirmed by mixed-effects models, attenuating the potential treatment effect. Notably, no on-treatment copeptin measurement was available between baseline and 7 months, precluding assessment of whether an early copeptin rise occurred and subsequently normalised. Our findings, however, are in line with the "acceleration-brake" hypothesis of Masuda et al. [26] , in which diuretic-induced volume depletion in response to prolonged SGLT2 inhibition is counterbalanced by AVP-mediated compensatory water reabsorption, establishing a new body-fluid steady state [26]. This interpretation is further supported by the DAPA-BODY trial, in which dapagliflozin increased copeptin by ~ 39% after one week but with normalised levels 6 months later, despite sustained fluid redistribution. Consistent with a transient volume response, a significant between-group difference in HCT emerged at 7 months in our population (44% vs. 42%, *p* = 0.03), resolving after withdrawal, whereas the overall treatment × time interaction was non-significant (*p* = 0.72), potentially reflecting the transient nature of the effect rather than its absence. That this difference was detectable for HCT but not copeptin at this sample size suggests haemoconcentration may be a more sensitive marker of SGLT2i-induced volume contraction than copeptin.

A further key distinction from prior research exploring SGLT2i-related effects on neurohormonal activation in populations at high cardiovascular risk is the absence of overt HF in our cohort (confirmed by normal CMR evaluated cardiac volumes and function). The EMMY trial [27] showed that empagliflozin initiated within 72 h of a large AMI reduced NT-proBNP and improved LV volumes and strain. In contrast, SOCOGAMI included individuals with smaller infarcts and normal left ventricular function, newly detected dysglycaemia without glucose-lowering medication, and SGLT2i initiation 6–10 weeks after the coronary event. This design isolates the neurohormonal response from acute-phase cardiac remodeling and mitigates pharmacological confounding. Copeptin is markedly elevated in both acute and chronic HF through compensatory AVP upregulation [28–30]. Marton et al. [24] reported mean copeptin levels at 16.0 ± 16.0 pmol/L in stable chronic HF with reduced ejection fraction *vs.* 4.9 ± 3.3 pmol/L in healthy controls (*p* < 0.001). Excluding HF patients in our study removed this confounder but may also have made a treatment effect harder to detect. Furthermore, although MI triggers an acute copeptin increase, this normalizes within hours [31]. A sustained neurohormonal activation beyond the first weeks is only documented in patients with overt HF or significant LV dysfunction [32]. Though subtle residual autonomic alterations may have contributed to the observed inter-individual variability in our cohort, baseline copeptin values (~ 7 pmol/L) measured 6–10 weeks after the coronary event were consistent with age- and sex-matched reference ranges, suggesting that the acute surge had resolved.

Copeptin correlated moderately with aPWV (rs = 0.40, *p* = 0.03), a recognized marker of subclinical cardiovascular disease and HF with preserved ejection fraction in diabetes [33]. The association is biologically plausible and consistent with prior studies linking copeptin to arterial stiffness and adverse cardiovascular outcomes [34, 35]. However, given the small sample size, the imprecise effect-size estimate, and potential sensitivity to aortic atherosclerosis, this finding should be regarded as exploratory and necessitates confirmation in larger cohorts. A baseline smoking imbalance (32% placebo vs. 5% empagliflozin), which may inflate this association, is a further reason for caution.

A key secondary aim of this study was to investigate the copeptin-glucose metabolism relationship in a stable post-ACS dysglycaemic setting. A maladaptive activation of the AVP-system has been linked to metabolic syndrome components, including insulin resistance and diabetes via the role of AVP in insulin and glucagon secretion through V1b receptors [36]. In the present analysis, copeptin levels neither correlated with glycaemic control nor predicted the glucose-lowering effect of the treatment. Copeptin increased slightly during the OGTT but with no statistically significant differences between treatment or glycaemic groups. The modest OGTT-associated rise in copeptin levels was driven by a responder subgroup rather than a uniform response (Supplementary Fig. 2), with a proportion that did not differ between IGT and T2DM (7 [39%] vs. 5 [33%], *p* = 1.0). This heterogeneity may relate to vasopressinase activity in insulin-sensitive tissues during the post-load state, raising copeptin secretion independently of a primary osmotic stimulus [37]. Further, one could speculate that AVP release may be more closely linked to cardiovascular stress and fluid balance than dysglycaemia per se in early dysglycaemic states. Due to limited power this finding remains hypothesis generating and its interpretation speculative, requiring dedicated investigation.

### Strengths and weaknesses

The double-blind, randomized design of SOCOGAMI limited confounding, and assessments at three timepoints, including one after treatment withdrawal, provided novel insight into potential rebound effects of SGLT2i therapy while also increasing statistical power through within-subject correlations. In addition, the absence of concurrent glucose-lowering medications throughout the entire study duration ensured that the glucometabolic effects were solely attributable to SGLT2 inhibition.

The present study does also contain some limitations. First, the lack of a statistically significant sustained effect on copeptin elevation may reflect a combination of limited power together with population- and design-related differences. Further, the sample size was small, causing some correlations to potentially be underestimated due to a type 2 statistical error: the observed 7-month difference, however, was 1.1 pmol/L (95% CI − 3.4 to + 5.7), an interval that includes the increases reported in comparable SGLT2i studies (~ 1.5–2.3 pmol/L) [16]. Accordingly, the non-significant result may reflect limited precision rather than evidence against an effect. The higher smoking prevalence in the placebo arm may also have biased copeptin upward in the placebo group, narrowing the between-group difference. Given the small numbers, however, this effect is likely minor relative to the study's limited power. Second, the single-center Swedish setting, may limit generalizability to broader populations and different healthcare systems. Third, without data on plasma volume, extracellular or interstitial fluid volume, urine osmolality, or body composition, our fluid-homeostasis interpretation is necessarily indirect, with HCT as the only proxy for volume status. Fourth, despite randomization, baseline diuretic use was more frequent in the empagliflozin arm (35% vs. 14%). As diuretics influence volume status and copeptin, this is a potential confounder. Nevertheless, results were unchanged after adjustment for baseline diuretic use and dose changes, as well as in sensitivity analyses excluding diuretic-treated patients (interaction p non-significant in all models). Finally, the very small IGT and T2DM subgroup sizes, limits OGTT results interpretability and copeptin changes during OGTT between these groups should be regarded as exploratory.

## Conclusion

In conclusion, this exploratory post-hoc analysis was unable to detect a statistically significant effect of empagliflozin on copeptin levels in post-ACS patients with newly detected dysglycaemia, likely reflecting insufficient statistical power and/or the presence of adaptive physiological mechanisms that limit AVP sustained secretion. Adequately powered studies including repeated copeptin sampling with an early start are needed to elucidate whether AVP modulation plays a direct role in the known benefits of SGLT2i.

## Supplementary Information

Below is the link to the electronic supplementary material.

Supplementary Material 1.

## Data Availability

All data supporting the findings of this study are available within the paper and its Supplementary Information. Some or all datasets generated during and/or analyzed during the current study are not publicly available but are available from the corresponding author on reasonable request.

## Abbreviations

2 h-PG: 2-Hour post-load glucose
ACS: Acute coronary syndrome
AMI: Acute myocardial infarction
ANOVA: Analysis of variance
aPWV: Arterial pulse wave velocity
AVP: Arginine vasopressin
BMI: Body mass index
CHD: Coronary heart disease
CMR: Cardiac magnetic resonance imaging
ECLIA: Electrochemical luminescence immunoassay
ECV: Extracellular volume
eGFR: Estimated glomerular filtration rate
FPG: Fasting plasma glucose
HbA1c: Glycated haemoglobin A1c
HCT: Haematocrit
HF: Heart failure
HOMA-IR: Homeostatic model assessment of insulin resistance
hsCRP: High-sensitivity C-reactive protein
IGT: Impaired glucose tolerance
LDL-C: Low-density lipoprotein cholesterol
LVEF: Left ventricular ejection fraction
LVEDVi: Left ventricular end-diastolic volume index
LVESVi: Left ventricular end-systolic volume index
LVMi: Left ventricular mass index
LVSVi: Left ventricular stroke volume index
MCR: Metabolic clearance rate
MRA: Mineralocorticoid receptor antagonist
NT-proBNP: N-terminal pro-brain natriuretic peptide
OGTT: Oral glucose tolerance test
REML: Restricted maximum likelihood
SD: Standard deviation
SGLT2i: Sodium-glucose cotransporter-2 inhibitor(s)
T2DM: Type 2 diabetes mellitus
UA: Unstable angina
