# Supplementary material for: Effect of empagliflozin on copeptin levels in patients with recent acute coronary syndrome and newly detected dysglycaemia: a post-hoc analysis of the SOCOGAMI randomized controlled trial
Source: Cardiovasc Diabetol. 2026 Jul 28;25:212. doi: 10.1186/s12933-026-03312-y (PMC13411128; doi:10.1186/s12933-026-03312-y)
Supplement: Supplementary file 1 — Supplementary Material 1. [file 12933_2026_3312_MOESM1_ESM.docx]

**Supplementary Material**

**Response of Copeptin Levels to Empagliflozin in Patients with Recent Acute Coronary Syndrome and Newly Detected Dysglycaemia**

**
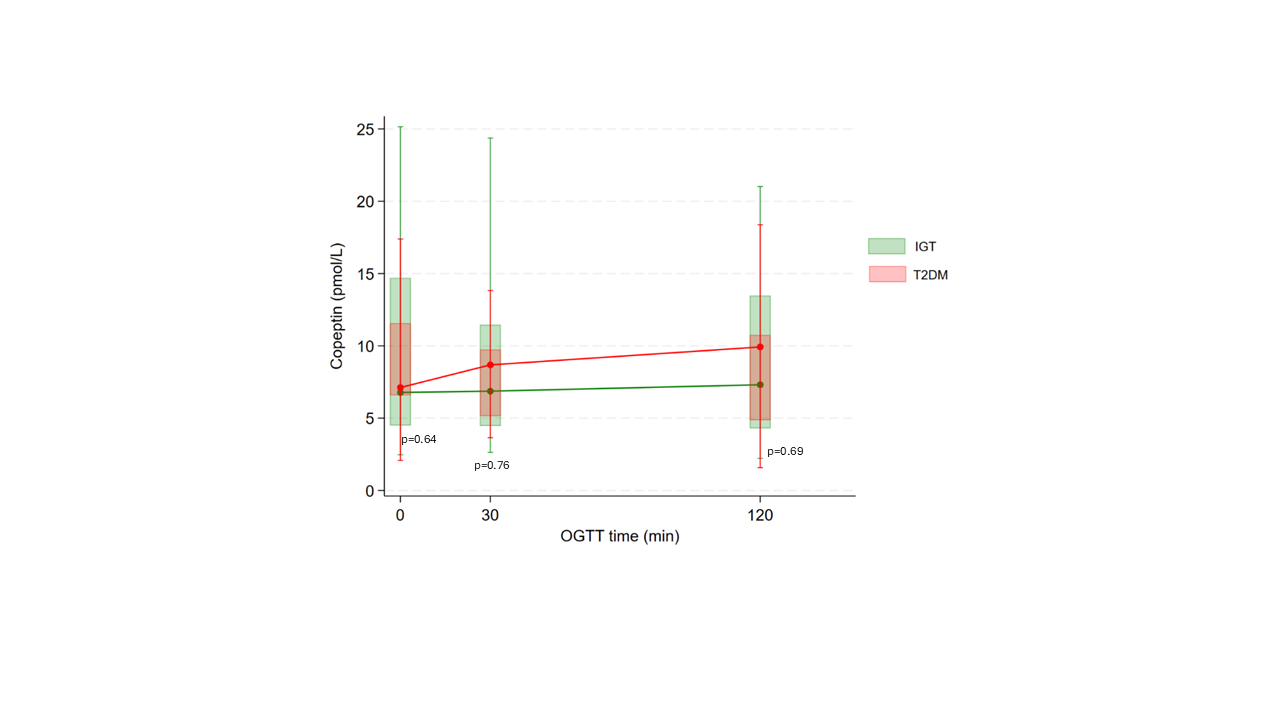
**

**Supplementary Figure 1** Copeptin levels during the OGTT at baseline by glycaemic group (empagliflozin and placebo group together).

Abbreviations: OGTT, oral glucose tolerance test; IGT, impaired glucose tolerance; T2DM, type 2 diabetes.

**
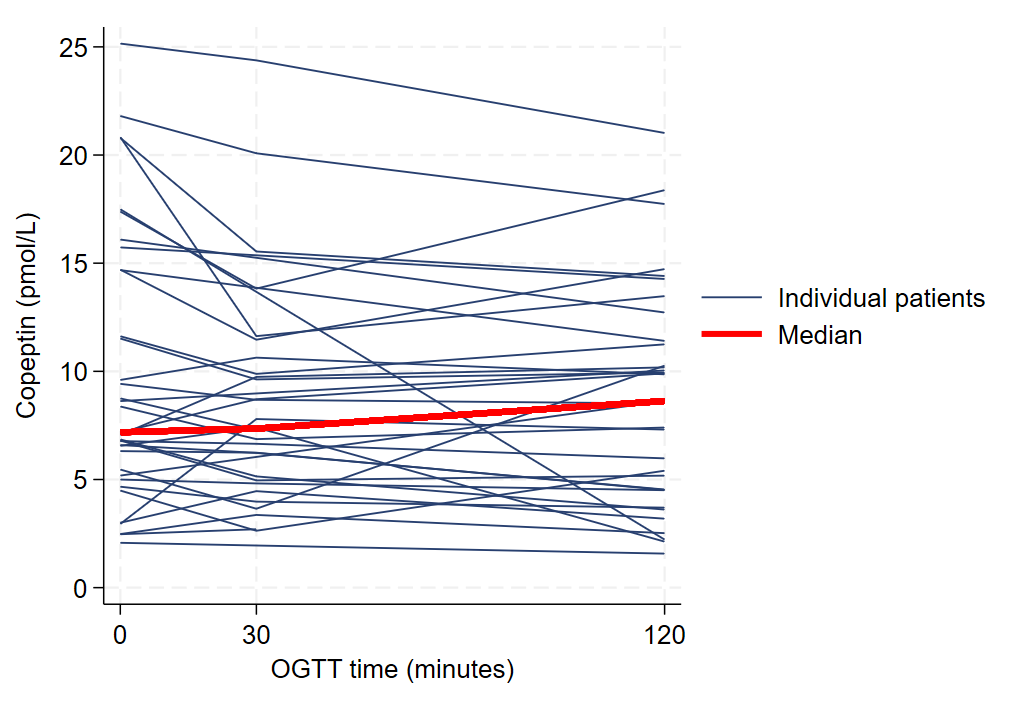
**

**Supplementary Figure 2.** Individual (N=35) copeptin trajectories during the baseline OGTT (0, 30, 120 min). Blue lines represent individual patients with at least two available OGTT measurements; the bold red line is the median trajectory


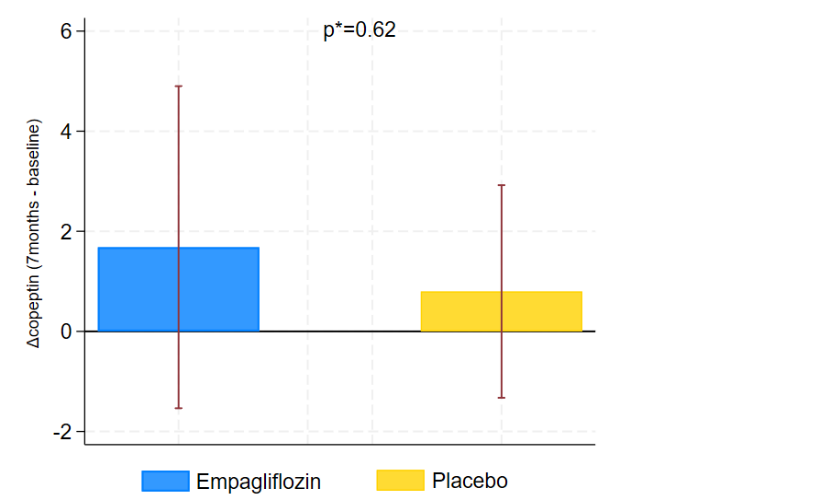


A)


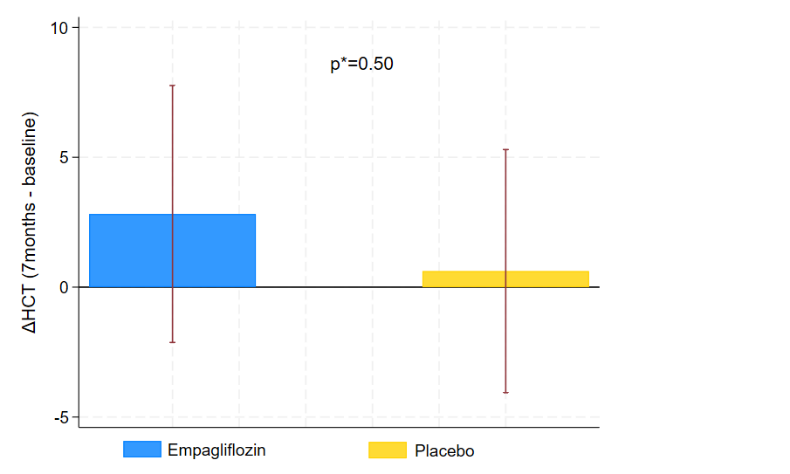


B)

**Supplementary Figure 3** Δ values of A) copeptin and B) haematocrit from baseline to after seven months on study drug. *P-values are by Mann-Whitney U Test.

Abbreviations: HCT, haematocrit.

**Supplementary Table 1. Copeptin data availability per visit and treatment group**

| **Visit** | **Empagliflozin (N=20)** | **Placebo (N=22)** | **Total (N=42)** |
| --- | --- | --- | --- |
| Baseline | 17 (85%) | 20 (91%) | 37 (88%) |
| 7 months | 18 (90%) | 19 (86%) | 37 (88%) |
| 10 months | 18 (90%) | 20 (91%) | 38 (90%) |
| **Total observations** | **53** | **59** | **112** |

*Data are N (%) of patients with available fasting copeptin at each visit. Missingness at baseline did not differ between arms (Fisher’s exact p=0.66). One empagliflozin patient had no copeptin data at any visit. Two placebo patients contributed a single observation each.*

**Supplementary Table 2** Evolution of the number of patients using diuretic agents and changes of diuretic doses throughout the entire follow-up period.

|  |  | **Empagliflozin** | | | | **Placebo** | | | | | |
| --- | --- | --- | --- | --- | --- | --- | --- | --- | --- | --- | --- |
|  | **Baseline** | **7 months** | | **10 months** | | **Baseline** | **7 months** | | **10 months** | |  |
| **Any diuretic agents** | 7 | 6 | | 6 | | 3 | 3 | | 3 | |  |
|  |  | **↑dose** | **↓ dose** | **↑dose** | **↓ dose** |  | **↑dose** | **↓ dose** | **↑dose** | **↓ dose** |  |
| **Loop/Thiazid** | 7 | 0 | 0 | 0 | 0 | 3 | 0 | 0 | 0 | 0 |  |
| **MRA** | 2 | 1 | 0 | 0 | 0 | 0 | 0 | 0 | 0 | 0 |  |
|  |  | **Empagliflozin** | | | | **Placebo** | | | | | |
|  | **Baseline** | **7 months** | | **10 months** | | **Baseline** | **7 months** | | **10 months** | | |
| **ACEis/ARBs** | 17 | 16 | | 16 | | 18 | 16 | | 16 | | |
| **β-Blockers** | 19 | 18 | | 18 | | 21 | 20 | | 20 | | |
| **Ca-Inhibitors** | 5 | 4 | | 4 | | 4 | 4 | | 4 | | |

Abbreviations: ACE, angiotensin converting enzyme; ARB, angiotensin receptor blocker; β-blockers, Beta blockers; Ca-blockers, Calcium channel blockers; MRA, Mineralcorticoid antagonists.

**Supplementary Table 3** Spearman correlations of copeptin changes from baseline and 7 months with insulin sensitivity indexes, hydro-electrolyte balance variables and cardiac magnetic resonance parameters, in the empagliflozin and placebo groups

|  | Δ**Copeptin** | | | |
| --- | --- | --- | --- | --- |
|  | **Empagliflozin** | | **Placebo** | |
| **Variable** | **r_s_** | **p-value** | **r_s_** | **p-value** |
| ΔFPG | -0.21 | 0.44 | 0.33 | 0.18 |
| Δ2h-PG | -0.02 | 0.93 | -0.15 | 0.54 |
| ΔHOMA-IR | 0.15 | 0.58 | -0.21 | 0.40 |
| ΔMCR Stumvoll | -0.08 | 0.79 | -0.28 | 0.26 |
| ΔHCT | 0.05 | 0.86 | 0.05 | 0.85 |
| ΔNa | 0.49 | 0.08 | 0.55 | 0.02 |
| ΔK | 0.12 | 0.67 | 0.45 | 0.06 |
| ΔLVEDVi | 0.02 | 0.17 | -0.16 | 0.53 |
| ΔLVESVi | -0.25 | 0.44 | -0.16 | 0.53 |
| ΔLVEF | 0.27 | 0.39 | -0.05 | 0.84 |
| ΔLVSVi | 0.30 | 0.34 | -0.10 | 0.70 |
| ΔLVM | 0.33 | 0.27 | 0.35 | 0.17 |
| ΔECV | 0.36 | 0.27 | 0.31 | 0.24 |
| ΔaPWV | -0.22 | 0.47 | 0.42 | 0.10 |

Abbreviations: FPG, fasting plasma glucose; 2h-PG, 2-hour post-load glucose; HOMA-IR, Homeostatic assessment model – Insulin Resistance); MCR, Metabolic clearance rate; LVSVi, left ventricular stroke volume indexed; LVEDVi, left ventricular end-diastolic volume indexed; LVESVi, left ventricular end-systolic volume indexed; LVEF, left ventricular ejection fraction; aPWV, arterial pulse wave velocity; ECV, extracellular volume; LVMi, left ventricular mass indexed; Na, Sodium; K, Potassium; HCT, haematocrit.

**Supplementary Table 4** Variation in copeptin levels between empagliflozin and placebo group during the OGTT throughout the entire study duration.

| **Copeptin** | **Empa** | **Placebo** | **P*** | **P_I_** |
| --- | --- | --- | --- | --- |
| **Baseline** |  |  |  |  |
| 0 min | 7.18 (5.46,11.62) | 6.72 (5.09, 15.21) | 0.78 |  |
| 30 min | 8.69 (4.96, 9.74) | 7.82 (4.51, 13,48) | 0.84 |  |
| 120 min | 9.21 (4.73,10.76) | 7.82 (4.51, 13.48) | 1.00 |  |
| **7 months** |  |  |  |  |
| 0 min | 11.51 (5.92, 16.3) | 8.42 (5.00, 19.01) | 0.63 |  |
| 30 min | 9.71 (5.70, 12.57) | 5.26 (7.38, 14.90) | 0.53 |  |
| 120 min | 10.70 (5.67, 13.17) | 7.01 (4.92, 16.37) | 0.39 |  |
| **10 months** |  |  |  |  |
| 0 min | 6.67 (5.36, 10.67) | 8.22 (5.94, 13.66) | 0.50 | 0.64 |
| 30 min | 5.65 (4.98, 9.00) | 6.88 (4.97, 10.12) | 0.66 | 0.23 |
| 120 min | 6.52 (5.66, 9.77) | 6.31 (4.58, 11.21) | 0.73 | 0.34 |

Data are median (Q1-Q3). P* = p-value by Mann-Whitney U-test. P_I_= P for interaction between treatment allocation and visit time (baseline, 7 months and 10 months) in the repeated measures ANOVA model after log transformation for each OGTT time point.

Abbreviations: OGTT, oral glucose tolerance test.
